# Supplementary material for: Are carbon emissions trading and green financial instruments synergistic? -Comprehensive quantitative research based on content analysis
Source: PLoS One. 2024 Mar 7;19(3):e0298601. doi: 10.1371/journal.pone.0298601 (PMC10919601; doi:10.1371/journal.pone.0298601)
Supplement: S1 Data — (ZIP) [file pone.0298601.s001.zip › Data/地级市Z_F工作报告.zip!/╜¡╦╒/─╧╛⌐╩╨/└┤╫╘╣1⁄2╓┌║┼í╛┬φ┐╦╩2╛▌═°í┐_2016.docx]

各位代表：

现在，我代表南京市人民政府，向大会报告工作，请予审议，并请政协委员和其他列席人员提出意见政府工作报告。

一、2015年工作回顾

过去的一年，我们认真贯彻党的十八大和十八届三中、四中、五中全会，以及习近平总书记系列重要讲话特别是视察江苏重要讲话精神，在市委正确领导下，紧紧围绕“四个全面”战略布局，主动适应经济新常态，加快建设“四个城市”，大力发展“五型经济”，全面展开建设“强富美高”新南京的伟大实践，统筹做好稳增长、促改革、调结构、重生态、惠民生和防风险各项工作，顺利完成了市十五届人大三次会议和“十二五”规划确定的主要目标任务。

经济发展稳中有进，质量效益稳步提升。认真落实国家和省稳增长调结构各项政策，制定出台《南京市加快推动经济发展迈上新台阶行动计划》等系列政策措施，注重消费、投资、出口协同拉动，经济保持平稳健康增长。预计全年实现地区生产总值9600亿元，增长9.2%，人均地区生产总值达1.8万美元。经济总量、人均地区生产总值在副省级城市中位次，分别由2010年第10位跃升为第6位、第7位跃升为第4位。一般公共预算收入跃上千亿元台阶，达到1020 亿元，同口径增长9.3%;社会消费品零售总额增长10.5%，均实现了五年翻番。电子商务交易额增长30%。完成服务外包执行额130亿美元，继续保持全国领先。规模以上工业增加值增长8.1%，在副省级城市中名列前茅;规模以上工业企业利税总额增长10%以上。

创新引领转型升级，结构调整步伐加快。坚持以创新为核心动力推动转型升级，协调推进创新型、服务型、枢纽型、开放型、生态型经济发展。创新驱动战略深入实施，以“一区两园”为核心载体，加快建设苏南国家自主创新示范区，全市科技进步贡献率达到61%以上，高新技术产业产值占规模以上工业总产值比重提高 1.5个百分点，全社会研发经费支出占地区生产总值比重、每万人发明专利拥有量均保持全省第一、跻身全国前列。向中小微企业发放科技创新券5500万元。出台“创业南京”人才计划，大力推进“大众创业、万众创新”，创办各类众创空间80家，省级以上孵化载体达到79家。产业结构持续优化，服务业增加值占地区生产总值比重达到57.3%，提高1.5个百分点，金融业、软件和信息服务业增加值增长16%以上，旅游业收入增长11%以上，文化产业增加值增长 14%。制定实施“中国制造2025”南京实施方案和南京“互联网+”实施方案，总投资292亿元的中电熊猫G108项目正式投产，智能装备制造、卫星应用、下一代信息网络产业产值增幅超过15%。支持实体经济发展，全年为企业减免税费81亿元。国家现代农业示范区加快建设，生物农业产值达300亿元。节能减排扎实推进，石化、钢铁等高耗能产业占工业比重下降了3.6个百分点，预计万元地区生产总值能耗下降6%，煤炭消耗下降1.5%，完成主要污染物“十二五”减排目标任务。

深化改革扩大开放，发展活力不断增强。七大类283项重点改革任务有序推进。深入推进简政放权，完成新一轮机构改革。公布行政审批、行政权力等10张清单，全面取消非行政许可事项。推进商事制度改革，全市新增市场主体和注册资本分别增长65.3%和60.7%。稳妥推进国企国资改革，完成17家市属国有企业混合所有制改革，42家企业关闭清算和双集中管理，22家企业(股权)从传统工商领域退出。医改试点及医药价格综合改革全面推进，57家公立医院同步实施药品“零差率”销售。打造对外开放新优势，开发园区加快转型升级，海峡两岸产业协同发展和创新试验区建设全面推进，南京综保区建设步伐加快，启运港退税政策落地实施，南京电子口岸一期投入运行。龙潭跨境电子商务产业园正式运营，南京成为全省首个跨境电子商务“进出兼通”城市。正式实施空港外国人72 小时过境免签政策，新开14条国际航线，铁路“中亚班列”开通运行。

完善功能提升品质，环境质量明显改观。城市空间布局不断优化，江北新区获批国家级新区，完成总体规划及近期建设规划，新区建设全面启动。河西城市新中心发展框架基本形成，中新南京生态科技岛、南部新城加快建设，大校场机场顺利搬迁，鼓楼滨江、铁北、燕子矶、铁心桥—西善桥、麒麟等片区改造全面实施。轨道交通实现网络化运营，地铁3号线建成通车，5条轨道交通线路同步建设，运营里程达到225公里，位居全国第4。扬子江隧道顺利建成，成为五年来建成的第 5条过江通道。模范西路改造、红山南路东延等一批城市道路工程加快建设，城市综合路网体系进一步健全。城市文化形象不断提升，建成侵华日军南京大屠杀遇难同胞纪念馆三期，汤山温泉旅游度假区成功创建国家级旅游度假区，牛首山文化旅游区、金陵大报恩寺遗址景区开放运行，圆满举办佛顶骨舍利供奉大典，成功举办首届南京国际马拉松赛。城乡环境面貌明显改观，街巷停车、交通秩序得到好转，交通拥堵指数从全国第21位下降至第28位。落实“门前三包”责任制，城市管护机制逐步健全。全面实施烟花爆竹禁放。江南、江北生活垃圾焚烧发电项目和餐厨垃圾处理厂建成运营，生活垃圾资源化处理实现新突破。优化调整生态红线保护区域，基本建成100个美丽乡村示范村和1000多平方公里示范区，绿化造林4.2万亩。污染治理力度持续加大，实施加强大气污染防治计划，大幅提高排污费征收标准，全面强化生态环境监管措施。2005年底前注册营运的黄标车全部淘汰。金陵石化及周边、大厂、梅山和长江大桥地区等四大片区工业布局调整有序展开。全年空气质量优良天数235天，较上年增加45天。PM2.5浓度比2013年下降26.9%，降幅居全省第一。城区人均公园绿地面积达到 14.98平方米，比2010年增加1.3平方米。建成区绿化覆盖率44.1%，居副省级城市前列。

全面发展社会事业，民生福祉持续增进。民生十件实事全面完成，城乡公共服务支出占一般公共预算支出比重达77.7%，预计城乡居民人均可支配收入分别增长8%和10%，城乡居民收入差距逐年缩小，人均纯收入低于6500元农户全部脱贫。全力促进创业就业，启动实施“创业助推行动”，培育自主创业者 1.6万人，带动就业12.3万人，全市新增城镇就业22.2万人，其中大学以上人员占比达57.2%。完善社会保障体系，顺利实现“五险”市级统筹，启动实施城镇职工和居民大病医疗保险，城乡低保和居民基础养老金实现全城同标。促进住房保障体系转型，推进公共租赁住房与廉租房并轨运行。新开工各类保障房 301万平方米，竣工296万平方米，累计有13.6万户居民入住保障房，完成城中村、危旧房改造254万平方米。优化提升公共服务，推进学前教育“增量、提优、普惠”工程，省、市优质园比例达到84%。完善义务教育学校招生办法，就近入学率超过90%。市公共卫生医疗中心、河西儿童医院投入使用，卫生服务体系健全率达到100%。推进“医养融合”，新增养老机构床位4771张。新增、更新公交车1243辆，新增公共自行车2.2万辆，主城区公交出行分担率达到50%，领先全国同类城市。人均拥有公共文化体育设施面积提高到2.8平方米。基本实现全市主要公共区域WIFI网络免费向公众开放。提高社会治理水平，新建40家街镇社区综合服务中心，扎实做好安全生产、食品药品和农产品质量安全、信访稳定、防汛应急处置等各项工作，妥善处置“东方之星”号客轮翻沉善后工作。平安南京、法治南京建设不断深化，公众安全感达93%。民族、宗教、双拥、外事、侨务、妇女、儿童、档案、史志、参事、仲裁、残疾人、慈善事业取得新业绩。

各位代表，2015年是“十二五”规划的收官之年。过去的五年，是我市经济发展实现历史性跨越、综合实力和竞争力显著提升的五年，是城市功能日臻完善、城市地位和影响力显著提升的五年，是创新和改革开放深入推进、城市发展活力显著提升的五年，是生态环境不断改善、城乡建设水平显著提升的五年，是各项社会事业快速发展、人民生活水平显著提升的五年。五年来，我们经历了一系列大事要事难事，有效应对了国际金融危机持续影响等风险挑战，成功举办了2013 年亚青会、2014年夏季青奥会，有效保障了南京大屠杀死难者国家公祭活动，经济社会发展取得了重大历史性成就。

过去五年取得的成绩，得益于历届市委市政府打下的坚实基础，得益于市人大市政协的倾力支持，是全市上下共同努力奋斗的结果。在此，我代表南京市人民政府，向在各行各业辛勤劳动的全体市民，向人大代表、政协委员、各民主党派、工商联和无党派人士、各人民团体、社会各界人士，向驻宁人民解放军、武警官兵，向所有支持南京建设与发展的香港、澳门特别行政区同胞，台湾同胞，海外侨胞和国际友人，表示衷心的感谢!

在肯定成绩的同时，我们也清醒地认识到，经济社会发展中还存在一些问题和挑战，正面对着深刻的供给侧、结构性和体制性矛盾。经济下行压力加大，产业结构仍然偏重，稳增长、调结构任重道远;科技、人才与产业结合度不够紧，创新能力还不够强，增长动力有待进一步转换;资源约束趋紧，节能减排压力加大，环境治理及城市长效管理任务艰巨;城乡区域发展不平衡，居民增收压力较大，就业、养老、教育、医疗、食品药品安全等公共服务水平有待进一步提升;制约发展的体制机制障碍仍然存在，国际化、市场化、法治化营商环境有待进一步完善。我们一定直面问题，切实增强忧患意识、责任意识，努力在破除瓶颈、补齐短板上取得明显进展。二、“十三五”发展的总体思路和目标任务

“十三五”时期，是南京大有作为的重要战略机遇期，但也面临着诸多矛盾叠加、风险隐患增多的严峻挑战。从国际形势看，世界经济在深度调整中曲折复苏，新一轮科技革命和产业变革正蓄势待发。同时，全球投资贸易增长持续乏力，外部环境中不确定不稳定因素增多。从国内形势看，我国经济长期向好的基本面没有变，新常态下的全国经济呈现出速度变化、结构优化、动力转换的新特点，正在向形态更高级、分工更精细、结构更合理的阶段演化。从我市情况看，当前正面临着 “一带一路”、长江经济带、长三角区域发展一体化等国家战略的重大机遇，以及国家级江北新区、苏南现代化建设示范区、苏南国家自主创新示范区建设的重要时机，为我们开辟了更大发展空间。我们必须科学判断和准确把握经济社会发展大势，充分认识新一轮发展面临的重大机遇，清醒认识面临的诸多挑战，坚定信心，锐意进取，攻坚克难，不断开创南京经济社会发展新局面。

根据市委关于制定南京市国民经济和社会发展第十三个五年规划的建议，市政府制定了“十三五”规划纲要(草案)，已经提请大会审议。南京“十三五”经济社会发展的总体思路是：高举中国特色社会主义伟大旗帜，全面贯彻党的十八大和十八届三中、四中、五中全会精神，以马克思列宁主义、毛泽东思想、邓小平理论、“三个代表”重要思想、科学发展观为指导，深入贯彻习近平总书记系列重要讲话特别是视察江苏重要讲话精神，紧紧围绕“四个全面”战略布局，牢记“两个率先”光荣使命，坚持现代化国际性人文绿都的城市定位，瞄准建设“一带一路”节点城市、长江经济带门户城市、长三角区域中心城市和国家创新型城市的奋斗目标，保持战略定力，坚持稳中求进，以提高发展质量和效益为中心，以推进“五个迈上新台阶”为重点任务，以对接落实全省“八项工程”为主要抓手，以发展创新型、服务型、枢纽型、开放型、生态型经济为主攻方向，统筹推进经济、政治、文化、社会、生态文明建设和党的建设，加快形成引领经济发展新常态的体制机制和发展方式，着力建设经济强、百姓富、环境美、社会文明程度高的新南京，确保率先全面建成小康社会，探索开启基本实现现代化新征程，在“建设新江苏”的奋进之路上走在前列、争当排头兵。

“十三五”经济社会发展的主要目标任务是：

——经济综合实力和国际竞争力明显提升。经济保持中高速增长，产业迈向中高端水平，地区生产总值年均增长8%左右，江北新区地区生产总值增速高于全市 5个百分点以上，提前实现全市地区生产总值和城乡居民人均收入比2010年翻一番。基本形成以服务经济为主的经济结构，服务业增加值占地区生产总值比重达到60%以上。新产业新业态不断成长，基本确立战略性新兴产业支柱地位，高新技术产业产值占规模以上工业总产值比重提高到52.5%。基本实现农业现代化，现代农业发展水平达到90%以上。基本形成全方位对内对外开放新格局，服务外包执行额年均增长10%，境外投资年均增长10%以上。初步确立“一带一路”重要节点城市地位，城市国际化水平大幅提升。

——科技创新能力和活力明显提高。全面建成国家创新型城市，主要创新指标达到创新型国家和地区中等以上水平。企业创新主体地位确立，“大众创业、万众创新”的体制机制更加健全，在江苏建设具有全球影响力的产业科技创新中心和有国际竞争力的先进制造业基地进程中走在前列。全社会研发经费支出占地区生产总值比重提高到3.2%，科技进步贡献率提高到65%。

——人民生活水平和质量普遍提高。居民人均可支配收入与全市经济同步增长，其中农村居民人均可支配收入增幅高于城镇居民1个百分点左右。中等收入群体持续扩大，符合标准的低收入人口全面脱贫、经济薄弱村全部摘帽。社会就业更加充分，就业层次持续提升，城镇登记失业率控制在4%以内，五年新增城镇就业 70万人。教育率先实现现代化，劳动年龄人口平均受教育年限达到13年。现代医疗卫生体系和健康名城建设取得更大进展，城乡居民主要健康指标达到国际先进水平。形成更加公平更可持续的社会保障制度，城乡基本社会保险覆盖率达到98%以上。推动住有所居向住有宜居迈进，城镇常住人口保障性住房覆盖率达到 23%以上。

——城乡区域协调发展水平显著提高。全面完成国家新型城镇化综合试点任务，新型城镇化和城乡发展一体化质量明显提升，城市空间格局和生产力布局更加合理，历史文化名城保护更加科学，现代基础设施体系更加完备，城市管理科学化水平显著提升，常住人口城镇化率达到83%，户籍人口城镇化率达到79%。长三角区域中心城市的辐射带动能力明显增强，长江经济带门户城市功能进一步健全。

——生态环境和宜居程度明显改善。生产方式、生活方式绿色低碳水平明显提升，环境风险得到有效控制，建设用地逐步实现减量化，主体功能区布局和生态安全屏障格局基本形成。绿色南京建设持续推进，生态产品供给持续增加，林木覆盖率达到30%。能源资源使用效率大幅提升，产业园区循环化改造基本完成。主要污染物排放总量大幅减少，空气质量达到二级标准的天数比例和地表水优于Ⅲ类水质的比例达到省定标准，基本实现污水全处理、城市生活垃圾分类收集全覆盖。生态文明制度体系更加健全，全社会环境意识显著增强。

——社会文明程度和社会治理能力显著提高。中国梦和社会主义核心价值观深入人心，个人品德显著提高，社会诚信体系基本建立。公共文化服务体系更加完善，文化事业和文化产业繁荣发展，文化产业增加值占地区生产总值比重达到8%以上，文化国际影响力持续扩大。公共安全水平明显提高，公众安全感保持在 92%以上。法治政府基本建立，城乡和谐社区建设达标率分别达到98%和96%，基本形成比较完善的国际化、市场化、法治化营商环境。率先在重要领域和关键环节改革上取得决定性成果，在全面深化改革中走在全省前列。

“十三五”规划纲要描绘了未来五年的宏伟蓝图，将成为市政府未来五年的施政依据，我们将以饱满的精神、充足的干劲、务实的作风，扎实推进各项目标任务的落实。我们坚信，在市委的正确领导下，经过全市人民的共同努力和不懈奋斗，“十三五”规划目标一定能够全面实现。三、2016年经济社会发展的主要任务

今年是“十三五”开局之年，做好今年工作至关重要。2016年经济社会发展主要指标是：全市地区生产总值增长8%以上，一般公共预算收入增长8.5% 左右，固定资产投资增长7%左右，社会消费品零售总额增长10%左右，外贸出口、利用外资保持正增长，服务业增加值占地区生产总值比重提高1个百分点，高新技术产业产值占规模以上工业总产值比重提高1.5个百分点，全社会研发经费支出占地区生产总值比重达到3.03%，全体居民人均可支配收入增长与经济增长同步，PM2.5浓度较2013年下降13%，单位地区生产总值能耗降幅、主要污染物排放强度达到省定标准，公众安全感保持在92%以上。

为实现以上目标，我们将认真贯彻中央、省、市委决策部署，切实践行“五大理念”，深入实施“六大战略”，大力发展“五型经济”，围绕结构性改革这一重点，全面落实去产能、去库存、去杠杆、降成本、补短板五大任务，着力稳定经济增长，着力推进转型升级，着力深化改革开放，着力保障改善民生，以“迈上新台阶、建设新南京”的优异成绩，实现“十三五”良好开局。今年我们将切实做好以下六个方面重点工作：

(一)大力实施创新驱动发展战略，加快构建现代产业体系

强化创新在发展中的核心地位，把发展的基点放在创新上，促进科技创新与“五型经济”紧密结合，加快构建以创新为主要引领和支撑的经济体系和发展方式。

发挥科技创新引领作用。围绕建设国家创新型城市和全球有影响力的产业科技创新中心重要基地，加快创新型经济发展。强化企业创新主体地位，支持规模以上企业建设研发机构，加快发展以企业为主体的产业技术创新战略联盟，培育壮大创新型企业集群。规模以上工业企业研发经费支出增长8%，建设1—2家市级重大公共技术服务平台，新建60家市级以上工程技术研究中心。提升创新载体建设水平，充分发挥“一区两园”示范引领作用，加快苏南国家自主创新示范区建设，推动开发园区向创新园区转型，加快发展众创空间。深入实施“创业南京”人才计划，创新人才服务方式，重点集聚10名科技顶尖专家，培育40名创新型企业家，引进480名高层次创业人才，引领3800名青年大学生创业。充分发挥高校和科研院所优势，加快推动科研成果就地转化，实现科技服务业总收入480亿元。

培育发展新动力。优化人才、技术、管理等创新要素配置，释放新需求，创造新供给，实现新发展。以创新释放消费需求，持续推进智慧城市、健康名城、畅游南京建设，用好国家内贸流通体制改革发展综合试点城市、国家电子商务示范城市建设等机遇，大力发展电子商务、文化创意等新模式、新业态，加快培育服务消费、信息消费、时尚消费、品质消费等新的消费增长点，提升消费对经济增长的贡献度。分类做好“去库存”工作，扩大普通住房土地供应，调整棚改货币化安置比例，逐步扩大公租房供应范围，有效释放住房刚性需求和改善性需求，促进房地产市场稳定健康发展。以创新优化投资结构，深化投融资体制改革，提高投资有效性，重点加大科技创新、产业转型、城市基础设施、生态环境、公共服务等领域投资，改进考核办法，完善奖惩机制，发挥投资对经济增长的关键作用。以创新提升出口水平，实施优进优出计划，大力发展新兴外贸业态，积极开展跨境电子商务进口试点，扩大自主品牌、高技术含量、高附加值产品出口，提升出口对增长的促进作用。大力支持实体经济发展，打好“降成本”组合拳，落实国家、省减税清费政策，清理各种不合理收费，切实降低企业经营成本。认真做好“去杠杆”工作，加强政府性债务管理，规范各类融资行为，遏制非法集资，加强风险监测预警，有效防范金融风险。

加快供给侧结构性改革。坚持“调高、调轻、调优、调强、调绿”的导向，推动产业高端化发展，提高全要素生产率，倒逼产业转型升级。落实“中国制造 2025”南京实施方案，全面实施企业制造装备升级计划、企业互联网化提升计划，加快构建现代产业体系。大力发展智能制造，加快实施智能工厂培育计划，前瞻性布局工业机器人、3D打印等智能制造技术，启动20家智能工厂创建工作，推进智能工厂示范城市建设。落实南京“互联网+”实施方案，促进以大数据、云计算为代表的信息技术与制造业、生产性服务业等融合创新。运用先进适用技术和现代信息技术，加快传统产业技术升级、设备更新改造。培育壮大战略性新兴产业，瞄准新一代信息技术、高端装备制造、新材料等七大类14个重点领域，加快推进LG新能源产业基地、中电熊猫电子装备产业基地、中航南京航空装备产业基地、中船海洋装备机电产业基地等重点项目建设，提高产业集聚规模，加快建设全国战略性新兴产业重要基地。推动服务业提档升级，大力发展总部经济、金融服务、智慧物流等高端服务业，加快发展软件和信息服务、科技研发等生产性服务业，服务业增加值增长10%左右，全力打造中国软件名城、长三角区域金融中心和长江区域性航运物流中心。扎实做好“去产能”工作，积极运用市场机制、经济手段和法治办法，继续化解钢铁、水泥等过剩产能，加快淘汰建材、铅蓄电池、铸造、化工等落后产能。启动南化转型发展实施计划，实施扬子石化延长产业链提质增效、金陵石化油品升级改造计划。围绕今后五年每年减少钢铁产能100万吨的目标，引导南钢、梅钢提升产品附加值，提高节能环保标准。

着力推进农业现代化。加快国家现代农业示范区建设，推动科技、人才、资金等要素向园区集聚。大力培育家庭农场、合作社等新型农业经营主体，农户参加规范的专业合作社比重达75%，完成新型职业农民培训1.5万人。提高农业物质装备和技术水平，加快农业科技创新应用，新增高标准农田10万亩、设施农业 3.5万亩，农业机械化水平达85.5%。促进农村产业融合发展，推动农业生产与农产品加工、流通、服务有机结合，休闲农业综合收入达50亿元。实施“互联网+农业”工程，积极培育一批农业电子商务平台、示范主体，农业电子商务销售额增长30%。

(二)加大改革开放力度，增强经济发展动能活力

全面对接“一带一路”、长江经济带、长三角区域发展一体化等国家战略，在深化改革、扩大开放中拓展发展新空间。

加快推进江北新区建设。围绕“三区一平台”战略定位，细化完善江北新区发展总体规划，加快实施一批行动计划、重大工程、关键项目，努力把江北新区建设成为新南京发展的新龙头、现代化国际性人文绿都的标志性区域。主攻创新发展，加快推进省产业技术研究院落户江北，规划建设一批大学科技产业创新园、综合型科创服务中心、科技服务城。加快产业培育，围绕构建智能制造、生命健康、新材料、高端装备制造和现代物流、科技服务“4+2”现代产业体系，重点打造集成电路、生物医药、新能源汽车3个千亿级产业集群。加快推进西坝、七坝等港口建设，加快集疏运体系和临港综合配套服务区建设，提升港口物流业综合功能。推动基础设施和环境建设，加快推进南京铁路北站、轨道交通、综合管廊等一批重大功能性项目规划建设，推进规划展示馆等重大公益性场馆建设。推动滨江、绿水湾等长江湿地重点生态工程建设，优化城乡环境，着力构建宜居新区。创新体制机制，深化行政管理、科技、财政金融等关键领域改革，全面增强新区发展的内生动力。

深入推动重点领域改革。深化经济体制改革，深入推进百优民营企业培育计划，鼓励发展民营资本控股的混合所有制企业，鼓励引导社会资本投向城市基础设施、金融、公共服务、农业等领域。推进小微金融、科技金融、互联网金融创新，更好地支持民营经济发展。深化国有企业改革，实施国有资本有序进退，清理退出、重组整合、创新发展一批国有企业，提高国有资本配置和运营效率，推动国资监管向以管资本为主转变。建立现代财政制度，继续推进预决算信息公开，除涉密部门外，实现部门预决算公开全覆盖，严格财政预算执行。统筹专项资金使用，盘活存量资金，优化支出结构，规范转移支付，强化专项资金绩效评价及应用。实现不动产统一登记发证。持续深化农村改革，做好国家级农村改革试验区工作，深化转变土地利用方式创新试点，推进农村“三地一产”确权登记颁证，完善农村产权交易市场。

提高对外开放发展水平。主动服务和融入国家开放发展战略总布局，提升开放层次，拓展开放领域，增创对外开放新优势。加快开放型经济转型升级，提升利用外资水平，突出引资、引技和引智相结合，推进服务业领域有序开放，鼓励外资企业总部机构和研发机构落户南京，新增跨国公司地区总部、投资性公司等外资功能性机构15家。鼓励轨道交通、电力装备等优势企业“走出去”，扩大海外投资。加快南京电子口岸二期工程建设。实施部分外国人144小时过境免签政策和境外旅客购物离境退税政策。推动开发园区转型发展、创新发展，积极推广复制自贸区改革试点经验，创新体制机制和运行模式，更好地集聚高端人才、高端技术、高端产业。积极申报自贸试验区，高水平建设海峡两岸产业协同发展和创新试验区，推动化工园绿色转型，推进溧水开发区创建国家级开发区，打造高能级的开放型经济平台。发展壮大枢纽型经济，全面落实空港、海港、高铁港三大枢纽经济区发展政策措施，强化重大基础设施建设，完善综合枢纽功能，推动空港枢纽经济区申报省级开发区和空港综合保税区，创建国家临空经济示范区。加强与长江沿岸中心城市合作交流，全面推进都市圈协同发展，加快宁镇扬同城化步伐。

(三)推进城乡区域协同发展，提升中心城市功能品质

尊重城市发展规律，统筹做好城市规划、建设、管理，切实提高城市发展持续性、宜居性。

优化城市空间布局。强化规划先导作用，统筹生产、生活、生态空间布局，推进经济社会发展规划、城乡规划、土地利用规划、环境保护规划“多规合一”，把经济社会发展目标任务统筹整合到一张蓝图、一个平台上，聚焦发展重点，集约利用资源，增强可持续发展能力。加快主城区空间整合，严控老城建设规模和开发强度。推进河西城市新中心发展，加速建设河西南部地区基础设施和功能配套项目，加快推进南部新城、麒麟生态科技园、中新南京生态科技岛等片区开发建设，推动土山机场搬迁，实现经济发展与空间开发同步转型。加强城市更新改造，研究制定城市更新实施方案，重点推动鼓楼滨江、铁北、燕子矶、铁心桥—西善桥、麒麟等成片改造，释放发展新空间。大力推进副城与新城建设，巩固提升东山、仙林两个副城建设，支持江宁建设主城南部中心，高标准建设交通、能源、信息等基础设施，高质量配套教育、医疗、文化、体育等公共服务设施，提高城市现代化水平。有序推进溧水、高淳核心区建设，打造生态与文化、产业与城市相融合的现代化新城。

完善提升城市功能。加快综合交通体系建设，实施南京市全国综合运输服务示范城市建设方案，开工建设高淳至溧阳高速公路，加快完善干线公路网络，推进 104国道南京北段、340省道等国省干线公路建设。加快建设长江五桥，启动长江大桥公路桥维修改造工程，完成中央北路干道整治、雨花路拓宽改造等工程，推进纬七路东进二期等一批道路建设。全力创建“公交都市”，续建地铁4号线一期、5号线、宁和线一期和宁高线二期高淳段、溧水段，启动地铁7号线和1号线北延工程。续建马群等一批综合换乘设施，新建改造31座公交场站。加强市政设施建设，新建、改造供水管道90公里。加强地下管线数字化管理，鼓励地下空间复合利用，有序推进地下综合管廊建设运营。全面推进海绵城市建设，结合江北新区核心区和南部新城开发建设，实施一批先导示范项目。

推进新型城镇化和新农村建设。深化新型城镇化综合试点和新市镇综合改革，重点推进谷里街道、横溪街道等12个重点新市镇建设，做好江宁街道、金牛湖街道全国发展改革试点城镇工作。加快配套基础设施建设，推动城乡公交服务向新市镇延伸。实施村庄环境改善提升行动，推进规划布点村生活污水处理设施全覆盖，改善乡村环境面貌。全面建设美丽乡村，重点打造200平方公里美丽乡村示范区。推进重大水利工程建设，完成长江新济洲河段河道整治，加快推进秦淮东河一期先导段、固城湖、石臼湖防洪能力提升等工程。

强化城市精细化管理。按照《南京市城市治理标准化实施方案》要求，完善区及部门管理标准体系建设，严格落实层级管理责任，实施科学考评，推动城市治理全覆盖、精细化、长效化。以迎接国家卫生城市复审为契机，深化环境综合整治，全面推进“门前三包”，加强停车秩序管理，规范户外广告设置，加强城中村、背街小巷等环境薄弱地区的专项整治，加强住宅区物业管理，全面提升城市整体形象。推进国家生活垃圾分类示范城市建设，开工建设城东废弃物处置中心、江北废弃物综合处理厂，建成200个垃圾分类示范小区，提高生活垃圾无害化、资源化、减量化处理能力。

(四)全面推进绿色低碳发展，打造美丽宜居新家园

贯彻绿色发展理念，深入实施生态文明建设工程，把建设美丽宜居人文绿都的各项举措落到实处。

加强生态环境建设。全面落实《南京生态文明建设规划》，加大主体功能区规划落实力度，加快建设国家生态文明示范区。严格生态红线区域保护，细化生态红线边界划分，加强监督管理考核，建立健全生态补偿机制，把生态红线保护要求落到实处。加强生态环境保护制度建设，深化排污许可和排污权交易改革，健全环境资源价格激励约束机制。实施绿色南京工程，重点推进滨江等生态绿色廊道、城市楔形绿地建设，林木覆盖率达到29.4%。

大力推动绿色发展。实施能源与水资源消耗、建设用地等总量与强度“双控”行动，促进资源节约集约利用。大力推进绿色清洁生产，推动钢铁、石化、建材等制造业绿色化改造，完成100项重点节能项目和100家企业清洁生产审核。继续推进四大片区工业布局调整，完成一批工业企业搬迁关停。大力推进绿色服务和消费，全面推进绿色低碳循环交通运输区域性试点城市建设，鼓励绿色出行，支持新能源汽车推广应用，加快电动汽车充电设施建设。推广绿色建筑和建材，完成 300万平方米绿色建筑建设。

铁腕整治环境污染。持续下大力气治理灰霾，严格落实控煤措施，大力整治工业废气、扬尘、机动车尾气等污染，加强餐饮污染、秸秆焚烧等各类面源污染整治管控，加强重污染天气的应急处置，不断改善大气环境质量。强化水环境综合整治，治理工业废水，加强黑臭河道整治，切实保护集中式饮用水源地。加快推进污水处理厂提标改造、中水回用、污泥处置和配套管网建设，建成江宁城北污水处理厂二期，全年完成排水达标区创建230个。强化土壤污染整治，开展土壤污染现状调查，加强农业面源污染防治和工业污染场地治理。提升危险废弃物处置能力，防控土地污染环境风险。强化环境执法管理，组建环保联合执法指挥中心，推进环境监管网格化管理，采取按日计罚、限停限产、司法联动等手段，严厉打击环境违法行为。

(五)展示文明南京魅力，推动文化建设迈上新台阶

坚持社会主义先进文化前进方向，遵循文化发展规律，推动经济文化融合发展，提升城市文明程度，增强文化软实力，为建设新南京凝聚强大的精神力量。

建设先进文化高地。大力培育和践行社会主义核心价值观，发展先进文化、创新传统文化、弘扬红色文化，深入推进公民道德建设，深化群众性精神文明创建，不断提升文明城市创建水平。以严格的措施保护好城市物质文化遗产和非物质文化遗产，以及历史街区、历史风貌区和历史名镇名村，推进重要近现代建筑和重要工业遗产的保护利用，加快中国明清城墙、海上丝绸之路联合申遗，推动侵华日军南京大屠杀遇难同胞纪念馆升级国家馆，举办好国家公祭活动。推进城墙博物馆、东吴博物馆、南唐博物馆等文博场馆建设。有序推进历史文化名人展示工程，办好第七届中国(南京)世界历史文化名城博览会，展示南京历史文化名城魅力。

繁荣发展文化事业。完善公共文化服务体系，出台推进现代公共文化服务体系建设的实施意见，建设100个达到省级标准的社区综合性文化服务中心，推进江宁区创建国家级公共文化服务体系示范区。深入实施文化惠民工程，建立送书、送演出、送电影、送展览等文化下乡制度化机制，举办好“2016百场公益演出广场行”活动。推进全市公共图书馆“通借通还”一卡通服务。实施农家书屋提升工程，新建星级示范书屋50家，新增全民阅读新空间20个，积极创建首批江苏书香城市。实施文化精品创作工程，创作更多的优秀文艺作品，激发社会正能量，丰富市民精神文化生活。

促进文化产业提质增效。加快文化产业结构调整，促进文化与科技、金融、旅游、教育等全方位融合，培育移动游戏、数字教育等文化产业新业态，创建2—3 个示范性文化产业功能区，争创“国家文化金融合作试验区”。推进世茂梦工厂、华侨城文化旅游综合体、大明文化旅游度假区、长江路历史文化街等重点文化旅游项目建设，支持高淳国际慢城争创国家级生态旅游示范区和国家级旅游度假区。充分发挥南京创意设计中心、文化金融服务中心功能，基本建成“创意南京”融合公共服务平台体系。举办好第八届文化创意产业交易会等活动，文化产业增加值占地区生产总值比重达到6.3%。

(六)实施民生幸福工程，让人民群众共享发展成果

高度重视和加快改善民生，围绕“七个更”目标，扎实办好各项民生实事，全面提升群众的参与度、知晓度、满意度和获得感，努力在共享发展上走在全国全省前列。

全力促进就业创业和富民工作。深入实施居民收入倍增计划，建立完善更加积极的创业就业政策，多措并举持续增加城乡居民收入。把促进充分就业作为增加居民收入的重要途径，统筹推进高校毕业生、就业困难人员等重点群体就业，推行劳动者终身职业技能培训制度，城镇新增就业16万人，城镇登记失业率控制在 3.5%以内。健全科学的工资水平决定机制、正常增长机制、支付保障机制，完善企业工资集体协商制度。构建农民增收长效机制，通过鼓励农民创业就业、创新农业业态、激活农村产权权能等多种途径，促进农民收入持续较快增长，确保农村居民人均可支配收入增幅高于城镇居民1个百分点。

全面加快社会事业发展。提升教育整体质量，进一步优化幼儿园及中小学布局，持续推进学前教育普惠优质发展，实施“新优质初中”创建工程，义务教育优质资源覆盖率达85%。鼓励多元化办学，促进民办教育健康发展。加快创建南京软件科技大学。提升医疗卫生服务水平，深入推进公立医院管理体制改革，积极构建分级诊疗体系，推进医疗联合体、家庭医生制度、医养融合、智慧医疗建设，加快新城新区医疗资源配置，推进市中医院新院区、江北国际医疗中心建设。积极稳妥实施好“全面二孩”政策。加快全民健身设施建设，人均拥有公共体育设施面积提高到3.18平方米。举办好世界速度轮滑锦标赛等大型体育赛事。启动实施南京市回民殡葬服务所迁建。服务国防和军队改革，做好双拥共建工作。进一步做好工青妇、援疆援藏、侨务、档案、史志、地震、气象、社会科学等各项工作。

切实提升社会保障水平。坚持全覆盖、保基本、多层次、可持续方针，健全覆盖城乡居民的社会保障制度体系。着力提高综合保障水平，完善最低生活保障标准自然增长机制，同步提高农村五保、城市“三无”、困境儿童、特困残疾人等保障标准，确保与经济社会发展水平相适应。完善医疗救助体系，做好基本医疗保险、大病保险、医疗救助、慈善救助的相互衔接。实现临时救助常住人口全覆盖，为困难群众兜底线、救急难。强化安居保障，续建各类保障性住房800万平方米，新开工保障房400万平方米，竣工300万平方米。实施精准扶贫，做好新一轮低收入农户、经济薄弱村的脱贫帮扶工作，加大投入力度，落实年度目标任务，确保扶贫工作取得阶段性成果。

创新社会建设管理机制。深入开展全国社区治理和服务创新试验区建设，提档升级30家新型社区综合服务中心，城市和农村和谐社区建设合格率分别达到 94%和87%。落实社区工作准入制度，建立健全社区协商民主机制，深化社会组织登记制度改革，实施行业协会商会等四类社会组织直接登记。大力推进阳光信访，有效预防和化解矛盾纠纷。实行安全生产“党政同责、一岗双责”，加大监管执法力度，坚决预防和遏制重特大事故发生。启动市级灭火救援应急中心建设。全面推进国家食品安全城市创建工作，加大食品药品监管力度，保障市民饮食和用药安全。深入推进平安南京建设，确保社会和谐稳定。

今年市政府将重点办好民生十件实事：(1)促进就业创业。扶持大众自主创业1.3万人，带动就业8万人。援助困难人员就业1万人。开展各类职业技能培训30万人次。(2)方便城乡居民出行。新辟和调整公交线路30条以上。新建公共自行车服务网点380个以上，新增公共自行车1.45万辆以上。启动实施铁心桥立交等9个交通拥堵节点改造，缓解7条拥堵路段，改造红山南路等5条“断头路”。新改建农村公路400公里、农路桥梁16座。(3)提升医疗保障救助水平。城镇居民基本医保筹资标准提高到550—910元，其中老年居民财政补助标准提高到460元，其他居民、学生儿童、大学生财政补助标准提高到 430元。新农合筹资标准提高到850元，财政补助标准提高到620元。困难居民医保范围内自付部分救助比例提高到85%，取消门诊大病及住院救助封顶线，确保城乡14.3万最困难群众病有所医。(4)完善健康服务体系。全市医疗联合体核心医院每年下派不少于300名管理、医疗、护理、康复专家到基层医疗机构，实现专家指导帮扶全覆盖。重点人群家庭医生签约率达70%。为30万企业退休人员免费健康体检。为3万名以上市民免费体质检测。(5)提高养老服务保障水平。城乡居民基础养老金最低计发标准增加到每月345元。提高被征地人员老年生活困难补助标准，每月增发45元。建成1200个社区居家养老服务中心，其中由专业社会组织运营、具备“助餐、助医、助急”等养老服务能力的3A级社区居家养老服务中心达200个。全市80%以上街道至少设立一个中心厨房，助餐服务达到300万人次。(6)加强扶残济困。提高重度残疾人生活补贴和护理补贴标准，对无业无固定收入的三、四级智力和精神残疾人实施生活救助。对独生子女伤残、死亡特别扶助对象年满60周岁的，扶助金标准每人每月提高200元。城乡低保标准提高到750元。(7)改善教育薄弱环节。加大老城范围外教育配套力度，建成29所幼儿园、中小学校。增加接纳进城务工人员随迁子女学校的公用经费，改善民办新市民学校办学条件。增加困难学生资助经费，确保九类家庭经济困难学生享受政府助学。(8)改造棚户区、危旧房。对180个以上老旧小区进行综合整治出新，对200个老旧小区增设围墙封闭管理。完成主城六区286处老旧小区二次供水设施改造，惠及近10万户居民。完成250万平方米棚户区改造。(9)整治黑臭河道和积淹水片区。基本消除建成区36条河道黑臭现象。实施环陵路岔路口、汉中路等21处积淹水片区(点)改造。(10)实施脱贫增收工程。农村年人均可支配收入9000元以下的低收入人口，脱贫比例达到20%以上;20%的经济薄弱村实现稳定收入达百万元以上。四、全面提高政府治理现代化水平

过去一年，我们坚持依法行政、转变职能、改进作风，政府自身建设不断加强。制定实施《关于深入推进依法行政加快建设法治政府的意见》，把依法行政落实到政府工作全过程。健全完善市级执法监督、区级综合执法、镇街巡查监管新体制，实现严格规范、公正文明执法。加大行政监察和审计监督力度，市级98个部门全面公开部门预算、“三公”经费预算，市级公共财政、政府性基金、国有资本经营、社会保险基金预算实现审计全覆盖。强化目标责任落实，对年度293项重点目标任务实行“挂图作战”，确保按序时进度高效推进。自觉践行“三严三实”，坚决纠正“四风”问题，作风建设向纵深推进。强化党风廉政建设“一岗双责”，对慢作为、不作为、乱作为、失职渎职等行为严肃追责，对各类违纪违法案件严肃查处。办理市人大代表议案建议372件、市政协提案606件，满意率达 99%。

我们还清醒地认识到，政府自身建设仍存在许多不足。政府职能转变还未完全到位，依法行政能力有待提高，干事创业的精气神需要进一步提振，少数公务人员不勤不廉现象依然存在。对此，我们一定高度重视，切实采取有效措施，转变作风、提升效能，加强自身建设，加快推进政府治理体系与治理能力现代化。

深化法治政府建设。全面依法履行政府职能，制定实施法治政府建设规划，依法设定权力、行使权力、制约权力、监督权力，完善政府部门权力清单和责任清单制度，塑造守法、高效、廉洁的政府形象。健全科学民主决策机制，完善公众参与、专家论证、风险评估、合法性审查、集体讨论决定等重大行政决策程序。拓宽公民有序参与立法途径，所有法规规章草案公开征求意见，对涉及民生重要项目开展听证。推进重点领域行政执法体制改革，扩大相对集中处罚权范围，推进跨部门综合执法。建立部门执法责任清单，健全确责、履责、问责的执法责任体系。强化法治教育，弘扬社会主义法治精神，不断增强全体市民的法治观念。

强化政府执行能力。坚持主动作为，切实履行职责，狠抓贯彻落实，真正做到勤政为民。强化目标任务督查落实，加强监察执纪问责，加大对懒政、怠政、不作为等行为治理力度，依法依规严肃问责，推动各项工作提速、提质、提效。完善机关绩效管理工作办法，加快推进机关职能转变和公共管理创新。健全重大决策执行保障体系，推进重大政策举措第三方评估，探索建立“决策、督查、评估”三位一体工作推进机制。加强审计监督，推进公共资金、国有资产、国有资源和领导干部经济责任履行情况审计全覆盖。

切实提升服务效能。坚持联系群众、联系企业等各项制度，切实提高服务百姓、服务企业、服务发展的能力和水平。坚持简政放权、放管结合、优化服务协同推进，开展相对集中行政审批权改革试点，优化行政审批服务，进一步减程序、减费用、减时限，提高办事效率。整合优化投资项目并联审批流程，逐步向区(园区) 试点延伸。扩大政府购买公共服务，鼓励社会资本参与公益性事业投资运营，提高公共资源配置效率。深入推进“智慧政务”建设，启动建设市政务数据中心，推进政务外网扩容，进一步推动政府信息开放共享。提升12345政务热线服务质量，改进完善诉求办理和督查督办机制。

加强反腐倡廉建设。反腐倡廉建设永远在路上，反腐不能停步、不能放松。认真贯彻《廉洁自律准则》和《纪律处分条例》，切实践行“三严三实”，从严落实廉政建设“两个责任”，坚持把纪律挺在前面，强化权力运行制约和监督，健全惩治和预防腐败体系，做到干部清正、政府清廉、政治清明。完善作风建设长效机制，健全市、区、街镇三级作风监督网络，紧盯重要时间节点，紧盯“四风”新动向，紧盯群众反映强烈的干部作风突出问题，加大对群众身边“四风”和腐败问题的查处力度。加强行政权力监督，自觉接受人大及其常委会的法律监督与工作监督，自觉接受政协的民主监督，以及人民群众和社会舆论的监督。

各位代表，新的一年开启新的征程，新的征程承载新的梦想。“迈上新台阶、建设新南京”的光荣使命，鼓舞我们砥砺奋进;全市人民的信任与期盼，鞭策我们勇往直前。让我们紧密团结在以习近平同志为总书记的党中央周围，在省委省政府和市委的坚强领导下，同心同德，开拓创新，加快建设“强富美高”新南京，奋力谱写南京改革开放和现代化建设新篇章!
